# Severe corneal melting with necrotic sloughing in *Acanthamoeba* keratitis mimicking corneal endotheliitis

**DOI:** 10.1186/s12348-026-00614-w

**Published:** 2026-06-24

**Authors:** Yukihiko Nakajima, Hiroko Takano, Yuka Kondo, Ryo Taguchi, Machiko Shimmura-Tomita, Toshikatsu Kaburaki, Suguru Nakagawa

**Affiliations:** Department of Ophthalmology, Saitama Medical Center, Jichi Medical University, 1-847 Amanuma-cho, Omiya-ku, Saitama, 330-8503 Japan

**Keywords:** Acanthamoeba, Corneal endotheliitis, Keratic precipitates, Corneal melting, Necrotic sloughing, Corticosteroids, Contact lenses

## Abstract

**Background:**

*Acanthamoeba* keratitis (AK) is a sight-threatening corneal infection that may initially lack typical epithelial or perineural findings and mimic other infectious or inflammatory anterior segment disorders. Endotheliitis-like presentations of AK with stromal edema, keratic precipitates, Descemet membrane folds, and anterior chamber inflammation have been reported, but progression to severe stromal necrosis with spontaneous sloughing of necrotic corneal tissue is rarely documented.

**Case presentation:**

A 48-year-old contact lens wearer presented with right eye pain, redness, and decreased vision. Initial examination at a local clinic revealed anterior chamber inflammation without epithelial defects, radial keratoneuritis, or a ring infiltrate, and topical betamethasone was started before referral. At referral (0 weeks; 12 days after symptom onset), slit-lamp examination revealed diffuse stromal edema, fine keratic precipitates, Descemet membrane folds, and anterior chamber inflammation, closely mimicking viral corneal endotheliitis. Aqueous humor polymerase chain reaction for human herpesviruses 1–8 was negative. Because viral endotheliitis or noninfectious anterior uveitis was considered, topical corticosteroids and systemic prednisolone were administered (initially 30 mg/day and was gradually tapered). During follow-up, the clinical appearance evolved toward AK, and anti-infective therapy was revised. Corneal scraping was performed, but direct microscopy and bacterial and fungal cultures were negative. PCR testing of a corneal scraping specimen for *Acanthamoeba*, bacteria, and fungi was negative. Despite treatment, stromal necrosis progressed to severe corneal melting. At 34 weeks after referral, necrotic corneal tissue spontaneously sloughed as a sequestrum and was submitted for pathological evaluation. Hematoxylin and eosin and periodic acid-Schiff staining revealed necrotic corneal stromal tissue with inflammatory cell infiltration and multiple double-walled cystic structures consistent with *Acanthamoeba* cysts, confirming AK.

**Conclusions:**

This case illustrates a diagnostically challenging form of AK that initially mimicked corneal endotheliitis, yielded negative scraping-based microbiological tests, and was ultimately confirmed by histopathologic examination of spontaneously sloughed necrotic stromal tissue. Severe AK rarely manifests as necrotic stromal sequestration and spontaneous sloughing, in addition to the more commonly recognized destructive outcomes such as corneal thinning, perforation, or keratoplasty-requiring disease. Repeated diagnostic reassessment is important in contact lens wearers with atypical endotheliitis-like keratitis, particularly when the disease progresses despite antiviral or anti-inflammatory therapy.

## Background

*Acanthamoeba* keratitis (AK) is a potentially sight-threatening corneal infection strongly associated with contact lens wear. Typical findings include severe ocular pain, epithelial disease, superficial punctate keratitis, microcystic edema, radial keratoneuritis, multifocal stromal infiltrates, and ring infiltrates; however, AK may lack these hallmark signs early in its course and mimic other infectious or inflammatory anterior segment disorders [1, 2]. Previous reports have described AK presenting with stromal edema, keratic precipitates, and anterior chamber inflammation, thereby mimicking corneal endotheliitis despite an intact corneal epithelium at onset [3, 4]. Such presentations pose diagnostic challenges, because they may prompt initial consideration of viral endotheliitis or noninfectious anterior uveitis before the characteristic features of AK become evident.

The present case was clinically characterized by an endotheliitis-like presentation at referral, followed by severe stromal melting and spontaneous sloughing of necrotic corneal tissue as a sequestrum. Although repeated scraping-based microbiological tests, including microscopy, culture, and PCR, did not establish the diagnosis during the active course, histopathologic examination of the sloughed stromal tissue demonstrated multiple double-walled cysts consistent with *Acanthamoeba*. We report this clinicopathologic case to document this unusual destructive course and to emphasize the importance of repeated diagnostic reassessment in contact lens wearers with atypical anterior segment inflammation.

## Case presentation

A 48-year-old Japanese man who wore contact lenses developed right eye pain, redness, and decreased vision in the right eye at symptom onset (2 weeks before referral) and visited a local clinic 1 day later. Slit-lamp examination at the local clinic revealed anterior chamber inflammation without epithelial defects or a ring infiltrate. Because intraocular inflammation predominated despite an intact corneal epithelium, iritis was suspected, and topical betamethasone was prescribed. His symptoms did not improve, and corneal edema worsened. He was referred to our ophthalmology department 12 days after symptom onset.

**Fig. 1:**
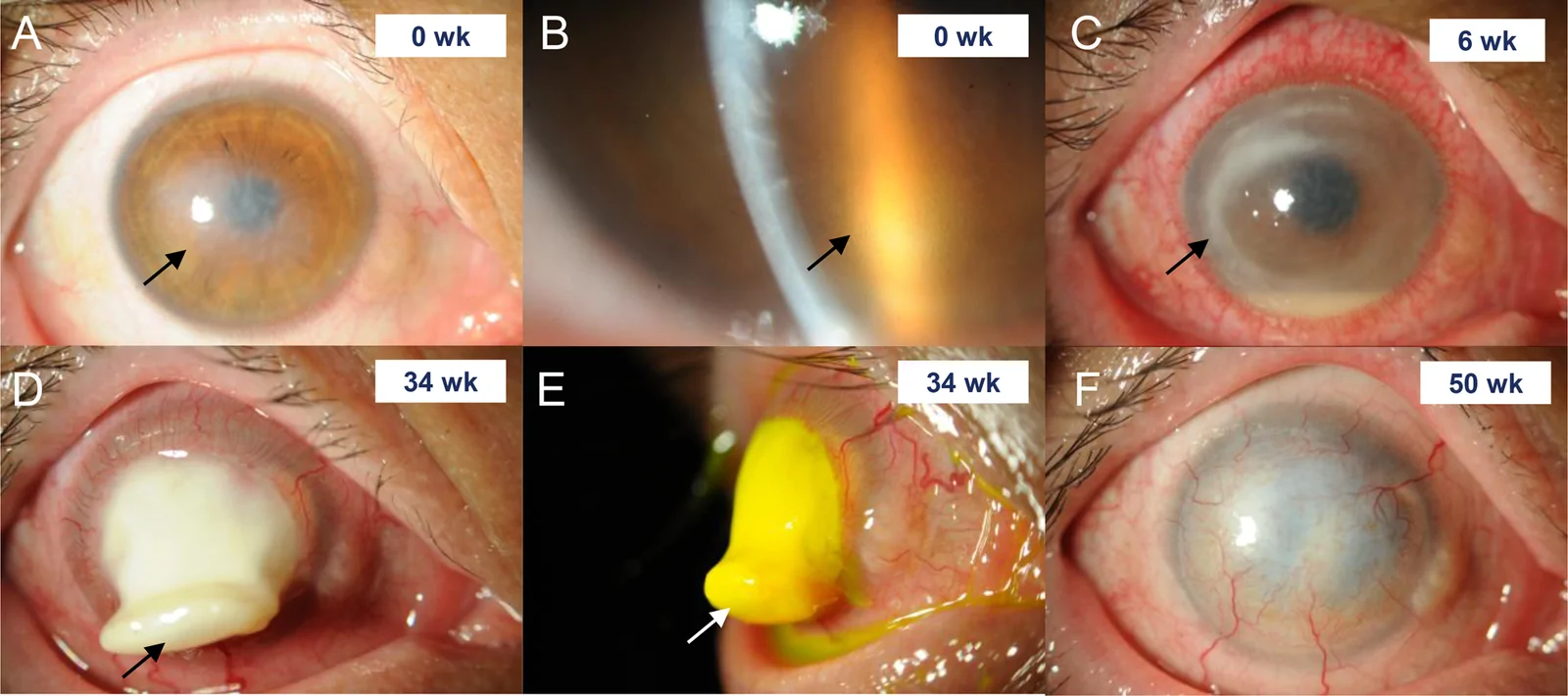
Representative slit-lamp photographs at key stages of disease progression. Timing labels within the panels indicate weeks after referral to our department. (**A, B**) At referral to our department (0 weeks), slit-lamp photographs showed diffuse stromal edema, corneal opacity, Descemet membrane folds, and fine keratic precipitates, simulating viral corneal endotheliitis. Arrows in (**A**) and (**B**) indicate corneal edema and opacity and fine keratic precipitates, respectively. (**C**) At 6 weeks after referral, a peripheral epithelial defect, progressive ring-like stromal infiltration, and hypopyon became evident, prompting clinical suspicion of *Acanthamoeba* keratitis. The arrow indicates the ring-like stromal infiltration. (**D, E**) At 34 weeks after referral, protruding necrotic corneal tissue consistent with sequestrum formation is shown in frontal and fluorescein-stained oblique views immediately before spontaneous sloughing; arrows indicate the protruding necrotic sequestrum. (**F**) At 1-year follow-up (50 weeks after referral), after spontaneous sloughing of the necrotic sequestrum, dense residual corneal opacity and marked corneal thinning remained

At referral (considered as 0 weeks), best-corrected visual acuity in the right eye was 0.02. Slit-lamp examination demonstrated diffuse stromal edema, corneal opacity, Descemet membrane folds, fine keratic precipitates, and anterior chamber inflammation, closely mimicking viral corneal endotheliitis (Fig. 1A, B). Epithelial defects, radial keratoneuritis, and a ring infiltrate were not clinically evident. Because topical corticosteroid had already been administered at the referring clinic, the fine keratic precipitates and anterior chamber inflammation may have represented a corticosteroid-modified inflammatory phenotype or secondary inflammatory deposition associated with deep stromal disease. Aqueous humor polymerase chain reaction for human herpesviruses 1–8 was performed because viral endotheliitis was considered; however, all results were negative. Topical betamethasone 0.1% six times daily, acyclovir 3% ophthalmic ointment five times daily, and tropicamide/phenylephrine once nightly were administered for presumed viral anterior uveitis or endotheliitis-like keratitis. Despite 1–2 weeks of topical acyclovir and empirical oral valganciclovir 900 mg twice daily from 4 to 6 weeks after referral, the corneal findings did not show improvement. An infectious etiology had not yet been confirmed; therefore, noninfectious anterior uveitis was also considered, and systemic prednisolone was initiated at 30 mg/day 3 weeks after referral.

The corticosteroid and topical medication course is summarized in Table 1, in which all time points are expressed in “weeks relative to referral to our department.” Prednisolone was tapered from 30 mg/day to 20 mg/day after 1 week and then to 10 mg/day. As corneal findings remained unresolved and *Acanthamoeba* keratitis was clinically suspected, the dose was further reduced to 5 mg/day and then 2.5 mg/day. Low-dose prednisolone was subsequently tapered with intermittent dose adjustments because of persistent inflammation and was discontinued 44 weeks following referral.

**Table 1:** Timeline of clinical course, diagnostic findings, and treatment

Time point relative to referral to our department	Clinical and diagnostic findings	Treatment
−2 weeks (symptom onset)	Right eye pain, redness, and decreased vision developed.	No treatment performed.
−2 weeks (first local clinic visit)	Anterior chamber inflammation without epithelial defect or ring infiltrate; iritis was suspected.	Topical betamethasone was initiated.
0 weeks (referral to our department)	Diffuse stromal edema, corneal opacity, Descemet membrane folds, fine keratic precipitates, and anterior chamber inflammation; no epithelial defect, radial keratoneuritis, or ring infiltrate. Aqueous humor PCR for human herpesviruses 1–8 was negative.	Topical betamethasone 0.1% six times daily, acyclovir 3% ophthalmic ointment five times daily, and tropicamide/phenylephrine once nightly were used for presumed viral anterior uveitis or endotheliitis-like keratitis.
3 weeks	Corneal opacity, Descemet membrane folds, and hypopyon worsened; noninfectious anterior uveitis was considered.	Oral prednisolone 30 mg/day was initiated. Topical betamethasone 0.1% was increased to eight times daily; acyclovir ointment was discontinued; tropicamide/phenylephrine four times daily, phenylephrine four times daily, and atropine 1% once daily were used.
4 weeks	Ciliary injection decreased and hypopyon disappeared, but corneal edema, Descemet membrane folds, and endothelial dysfunction-like findings persisted.	Oral prednisolone was reduced to 20 mg/day. Oral valganciclovir 900 mg twice daily (450-mg tablets, four tablets/day) was added empirically. Topical betamethasone 0.1% eight times daily, tropicamide/phenylephrine four times daily, phenylephrine four times daily, and atropine 1% once daily were continued.
5 weeks	No clear response to valganciclovir; hypopyon did not worsen; peripheral epithelial defect and stromal opacity became evident.	Oral prednisolone was reduced to 10 mg/day, and oral valganciclovir 900 mg twice daily was continued for an additional week. Topical betamethasone 0.1% was reduced to twice daily; tropicamide/phenylephrine twice daily and sodium hyaluronate 0.1% four times daily were used; phenylephrine and atropine were discontinued.
6 weeks	A peripheral epithelial defect, progressive ring-like stromal infiltration, and hypopyon became evident, prompting clinical suspicion of AK. Scraping from the epithelial defect was performed. Direct microscopy was negative. Fungal or mixed microbial keratitis also remained in the differential diagnosis.	Oral prednisolone was reduced from 10 mg/day to 5 mg/day. Oral valganciclovir was discontinued. Topical betamethasone 0.1% once daily and tropicamide/phenylephrine twice daily were continued. Pimaricin 1% ophthalmic ointment four times daily was initiated, and oral itraconazole 100 mg/day was started as empirical antifungal coverage, not as standard antiamoebic therapy.
7 weeks	Corneal epithelial scraping was repeated. No positive microbiological result was obtained from the scraping specimens available during this period.	Oral prednisolone 5 mg/day was continued. Topical corticosteroid was discontinued. Chlorhexidine 0.02% eye drops eight times daily, pimaricin 1% ophthalmic ointment four times daily, levofloxacin 1.5% eye drops four times daily, bromfenac eye drops twice daily, and tropicamide/phenylephrine twice daily were used; oral itraconazole 100 mg/day was continued.
8 weeks	Repeat corneal epithelial scraping was performed. Direct microscopy and bacterial and fungal cultures were negative. PCR testing for Acanthamoeba, bacterial, and fungal DNA using a corneal scraping specimen was also negative; calcofluor white staining was not performed on the scraping specimens.	Oral prednisolone was reduced from 5 mg/day to 2.5 mg/day. Chlorhexidine 0.02% eye drops eight times daily, pimaricin 1% ophthalmic ointment four times daily, levofloxacin 1.5% eye drops four times daily, bromfenac eye drops twice daily, tropicamide/phenylephrine twice daily, and oral itraconazole 100 mg/day were continued.
10 weeks	Ongoing keratitis treatment.	Oral prednisolone was reduced from 2.5 to 1.25 mg/day. Chlorhexidine 0.02% eye drops eight times daily, pimaricin 1% ophthalmic ointment four times daily, levofloxacin 1.5% eye drops four times daily, bromfenac eye drops twice daily, tropicamide/phenylephrine twice daily, and oral itraconazole 100 mg/day were continued.
11 weeks	Progressive dense whitish stromal infiltration, a large epithelial defect, and stromal melting were documented photographically. Fungal or mixed microbial keratitis was also considered.	Oral prednisolone was increased from 1.25 to 2.5 mg/day. Voriconazole 1% eye drops eight times daily were added as adjunctive empirical antifungal coverage. Chlorhexidine 0.02% eye drops eight times daily, pimaricin 1% ophthalmic ointment four times daily, levofloxacin 1.5% eye drops four times daily, bromfenac eye drops twice daily, tropicamide/phenylephrine twice daily, and oral itraconazole 100 mg/day were continued.
17 weeks	Persistent severe keratitis; mixed AK and fungal keratitis remained in the differential diagnosis.	Oral prednisolone was adjusted to 2.5 mg/day on 4 days per week and 1.25 mg/day on 3 days per week. Tropicamide/phenylephrine twice daily, pimaricin 1% ophthalmic ointment four times daily, fluoroquinolone antibacterial therapy (levofloxacin eye drops and/or ofloxacin ointment) four times daily, bromfenac eye drops twice daily, chlorhexidine 0.02% eye drops eight times daily, voriconazole 1% eye drops eight times daily, and oral itraconazole 100 mg/day were continued.
24 weeks	Gradual tapering continued.	Oral prednisolone was adjusted to 1.25 mg/day on 4 days per week and 2.5 mg/day on 3 days per week. Tropicamide/phenylephrine twice daily, pimaricin 1% ophthalmic ointment four times daily, fluoroquinolone antibacterial therapy (levofloxacin eye drops and/or ofloxacin ointment) four times daily, bromfenac eye drops twice daily, chlorhexidine 0.02% eye drops eight times daily, voriconazole 1% eye drops eight times daily, and oral itraconazole 100 mg/day were continued.
30 weeks	Persistent keratitis; repeat scraping was performed because posterior opacity and mixed infection were considered.	Oral prednisolone was continued at an alternating low-dose schedule. Pimaricin, fluoroquinolone antibacterial therapy, bromfenac, chlorhexidine, voriconazole, and oral itraconazole were continued.
34 weeks	Necrotic corneal tissue spontaneously sloughed as a sequestrum and was submitted for histopathology.	Topical anti-infective and adjunctive treatments were continued during the post-sloughing phase.
35 weeks	Corneal epithelialization was noted.	Oral prednisolone was reduced to 1 mg/day. Pimaricin, fluoroquinolone antibacterial therapy, bromfenac, chlorhexidine, and voriconazole were continued.
37 weeks	Corneal epithelialization was maintained.	Oral prednisolone was reduced to 0.5 mg/day; topical anti-infective and adjunctive medications were continued.
40 weeks	Congestion decreased despite tapering.	Oral prednisolone was reduced from 0.5 mg/day to 0.25 mg/day. Pimaricin, fluoroquinolone antibacterial therapy, bromfenac, chlorhexidine, and voriconazole were continued.
44 weeks	Corneal epithelialization and no obvious worsening.	Oral prednisolone 0.25 mg/day was discontinued. Chlorhexidine and voriconazole were reduced to six times daily; pimaricin, ofloxacin ointment once nightly, and bromfenac were continued.
50 weeks (1-year follow-up)	Globe preserved; dense corneal opacity and marked thinning remained; the anterior chamber and lens status could not be clearly assessed because of poor visibility through the dense corneal opacity; final visual acuity was hand motion; intraocular pressure was 15 mmHg.	Chlorhexidine and voriconazole were tapered to twice daily; bromfenac was continued. Pimaricin ointment was still being used at three times daily, and fluoroquinolone antibacterial therapy was discontinued. Immediate keratoplasty was not planned because of high surgical risk.

Between 5 and 8 weeks after referral, the clinical appearance evolved toward AK. At 6 weeks after referral, a peripheral epithelial defect, progressive ring-like stromal infiltration, and hypopyon became evident, prompting clinical suspicion of AK. Corneal scraping was performed repeatedly. Direct microscopy of the scraping specimens did not reveal organisms, and bacterial and fungal cultures were negative; calcofluor white staining was not performed on the scraping specimens. PCR testing for *Acanthamoeba*, bacterial, and fungal DNA was performed once using a corneal scraping specimen, and all results were negative. In vivo confocal microscopy was not performed.　Although microbiological confirmation was not obtained from the scraping specimens, AK was clinically suspected based on the characteristic clinical course.

Chlorhexidine 0.02% eye drops were initiated eight times daily. Pimaricin 1% ophthalmic ointment was administered four times daily, and oral itraconazole 100 mg/day was prescribed for empirical antifungal coverage given that fungal or mixed microbial keratitis could not be completely excluded.

Adjunctive antibacterial therapy with fluoroquinolones was also used, consisting initially of levofloxacin 1.5% eye drops, followed by levofloxacin eye drops and/or ofloxacin ointment depending on the clinical situation. Topical corticosteroids were discontinued.

**Fig. 2:**
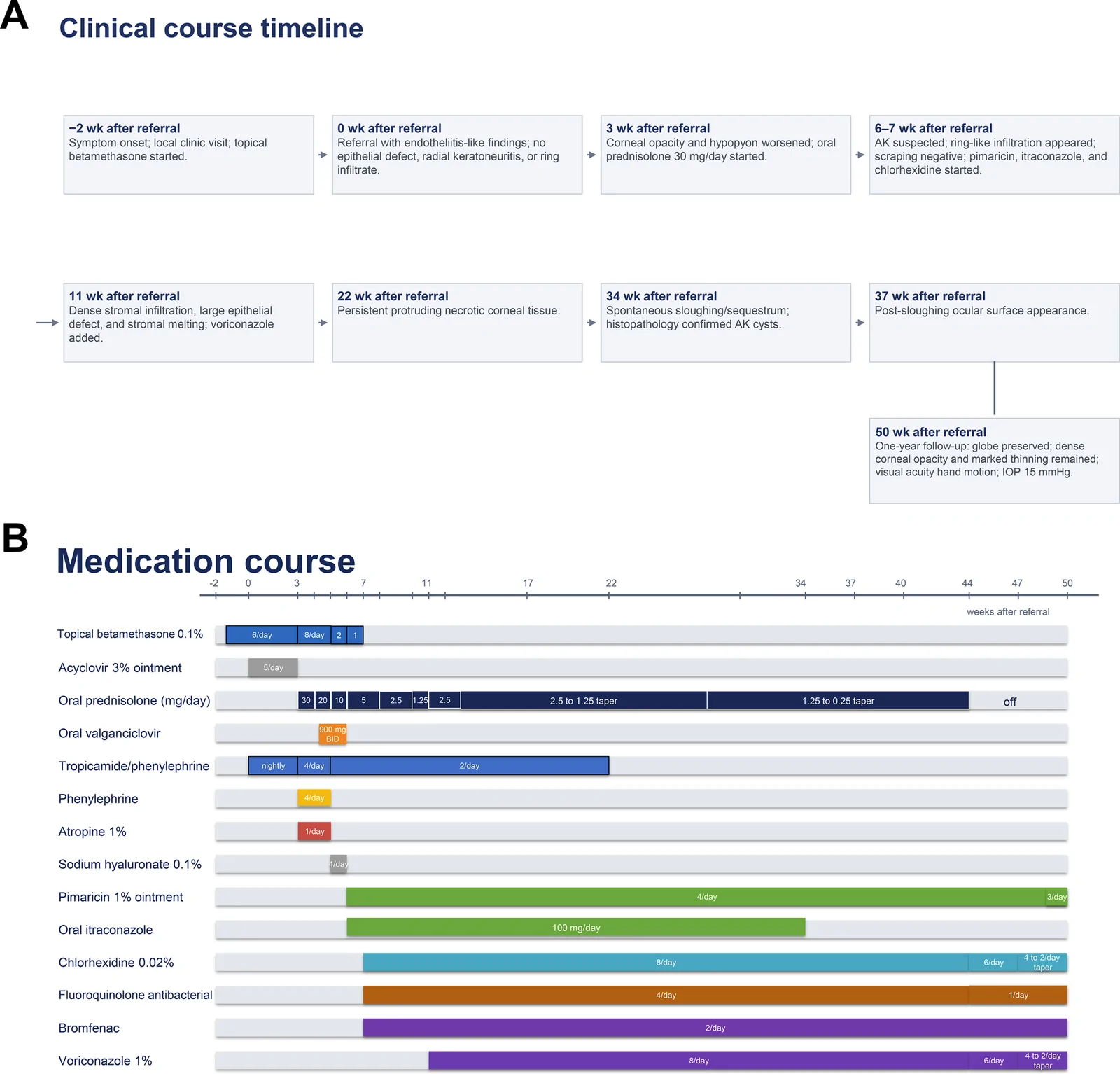
Timeline of the clinical course and medication course. In both panels, time is expressed in weeks relative to referral to our department. Symptom onset and the first local clinic visit are shown as negative time points before referral. (**A**) Clinical course timeline. The timeline summarizes symptom onset and the first local clinic visit, referral with endotheliitis-like findings, initiation of topical corticosteroid therapy before referral, initiation of systemic corticosteroid therapy, clinical suspicion of *Acanthamoeba* keratitis with appearance of ring-like stromal infiltration and hypopyon, initiation of chlorhexidine, onset of stromal necrosis and melting, protruding necrotic tissue/sequestrum formation, spontaneous sloughing, histopathologic confirmation of *Acanthamoeba* cysts, and the 1-year follow-up at 50 weeks after referral. The spontaneous sloughing/histopathologic confirmation milestone is fixed at 34 weeks after referral. (**B**) Medication course. Horizontal bars indicate the approximate duration and major dose or frequency changes of systemic and topical medications on the same weeks-after-referral axis. Oral valganciclovir was administered from 4 to 6 weeks after referral. Fluoroquinolone antibacterial therapy included levofloxacin 1.5% eye drops and/or ofloxacin ointment according to the clinical situation. For medications documented as continued or tapered after spontaneous sloughing, the bars extend to the 1-year follow-up at 50 weeks after referral; pimaricin ointment was reduced to three times daily at 50 weeks

**Fig. 3:**
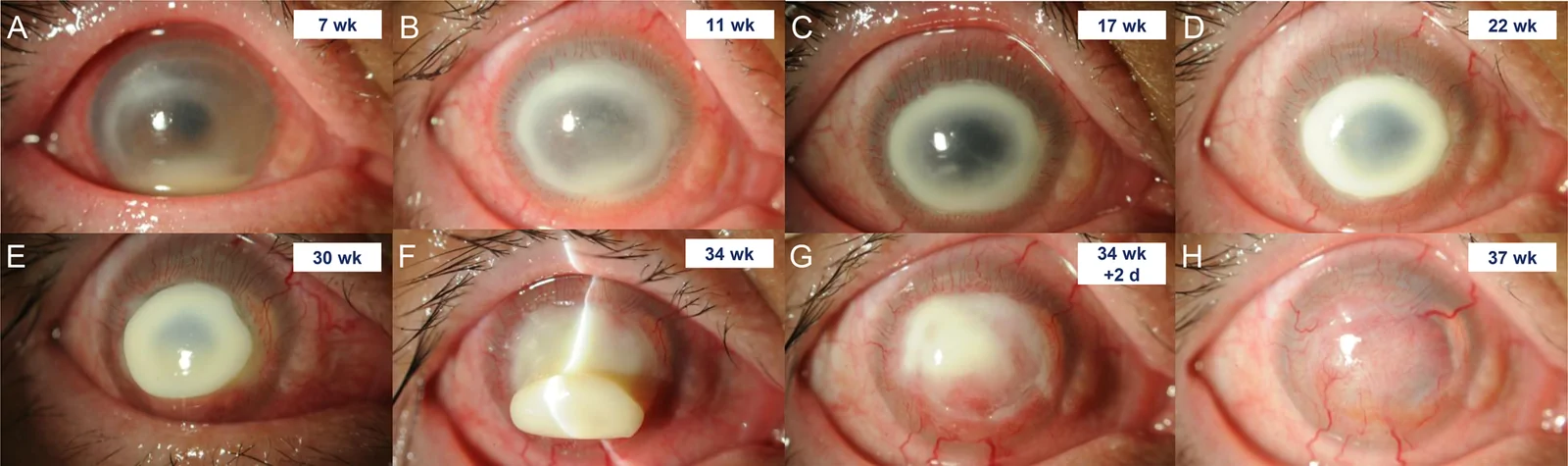
Serial anterior segment photographs showing progressive corneal destruction after clinical suspicion of *Acanthamoeba* keratitis. Timing labels within the panels indicate weeks after referral to our department. The 6-week photograph corresponding to the initial clinical suspicion of *Acanthamoeba* keratitis is shown in Fig. 1C. (**A**) At 7 weeks after referral, persistent stromal opacity and hypopyon were observed. (**B**) At 11 weeks after referral, dense stromal infiltration and progressive corneal opacity were present, corresponding to the phase of marked stromal melting summarized in Fig. 2A. (**C**) At 17 weeks after referral, circumscribed dense stromal opacity persisted. (**D**) At 22 weeks after referral, protruding necrotic corneal tissue became clinically apparent. (**E**) At 30 weeks after referral, further protrusion of necrotic corneal tissue was noted. (**F**) At 34 weeks after referral, prominent protruding necrotic tissue consistent with a sequestrum was present immediately before spontaneous sloughing. (**G**) At 34 weeks after referral, the ocular surface appearance two days after spontaneous sloughing of the necrotic sequestrum is shown; this is labeled as 34 weeks + 2 days in the figure. (**H**) At 37 weeks after referral, the post-sloughing ocular surface appearance showed dense residual opacity and marked corneal thinning

At 11 weeks after referral, slit-lamp photography showed progressive dense whitish stromal infiltration with a large epithelial defect and stromal melting, as summarized in the clinical course timeline (Fig. 2A) and illustrated by the serial anterior segment photograph obtained during this phase (Fig. 3B). Because the disease continued to worsen and fungal or mixed microbial keratitis remained clinically difficult to exclude, topical voriconazole 1% eye drops was added eight times daily as adjunctive empirical antifungal coverage.

**Fig. 4:**
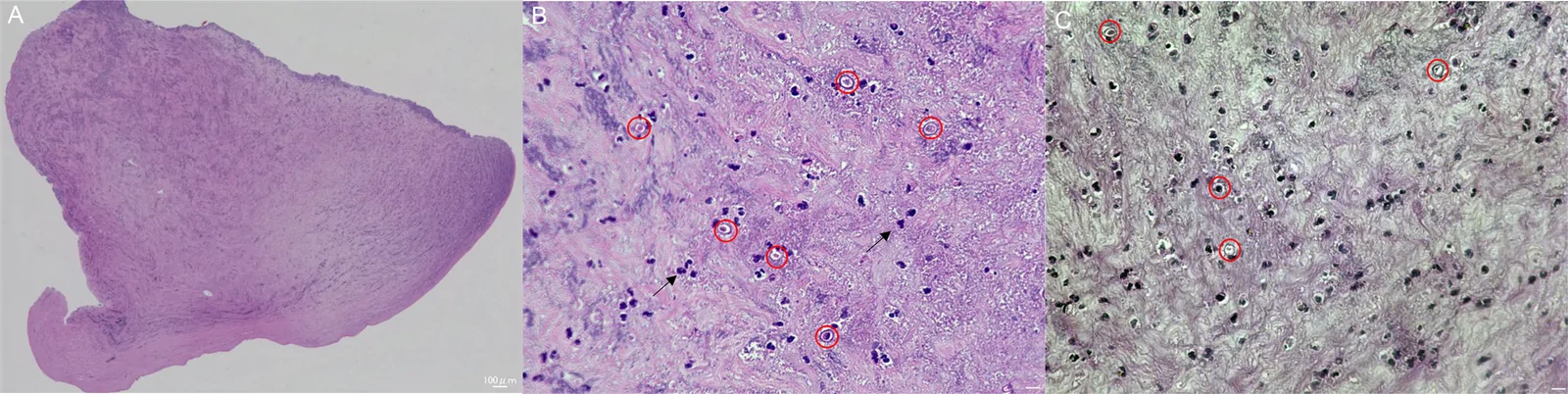
Histopathologic findings of the spontaneously sloughed necrotic corneal tissue obtained at 34 weeks after referral. (**A**) Low-magnification hematoxylin and eosin staining showed a sloughed necrotic corneal stromal fragment in which the normal lamellar stromal architecture was not clearly preserved throughout the examined tissue. (**B**) High-magnification hematoxylin and eosin staining demonstrated multiple round-to-oval double-walled cystic structures within eosinophilic necrotic stromal tissue with inflammatory cell infiltration. Red circles indicate representative cystic structures consistent with *Acanthamoeba* cysts, and black arrows indicate representative inflammatory cells. (**C**) High-magnification periodic acid-Schiff staining highlighted representative cyst wall-like structures consistent with *Acanthamoeba* cysts. Red circles indicate representative cystic structures. Because the examined specimen was a spontaneously sloughed stromal fragment rather than a keratoplasty specimen, and because identifiable corneal epithelium, the Descemet membrane, and corneal endothelium were not observed in the specimen, precise anteroposterior localization of the cysts and assessment of Descemet membrane penetration or endothelial invasion were not possible

Despite treatment, stromal necrosis progressed, resulting in severe corneal melting. At 34 weeks after referral, necrotic corneal tissue protruded as a sequestrum, which subsequently sloughed and was submitted for pathological evaluation (Fig. 1D, E; Fig. 3F, G). Histopathologic examination with hematoxylin and eosin and periodic acid-Schiff staining revealed that the specimen consisted of spontaneously sloughed necrotic corneal stromal tissue. On low-magnification hematoxylin and eosin staining, the normal lamellar stromal architecture was not clearly preserved throughout the examined specimen (Fig. 4A). On higher magnification, eosinophilic necrotic stromal material with inflammatory cell infiltration was observed, and multiple round-to-oval double-walled cystic structures consistent with *Acanthamoeba* cysts were identified within the necrotic stromal matrix (Fig. 4B). Periodic acid-Schiff staining highlighted cyst wall-like structures, further supporting their interpretation as *Acanthamoeba* cysts (Fig. 4C). No obvious fungal hyphae were identified in the examined hematoxylin and eosin or periodic acid-Schiff-stained sections. Grocott methenamine silver, Gram, and calcofluor white staining, as well as immunohistochemical staining, were not performed on the histopathologic specimen. The examined specimen consisted of a spontaneously sloughed necrotic stromal fragment rather than an excised keratoplasty button. Because identifiable corneal epithelium, the Descemet membrane, and corneal endothelium were not observed in the specimen, precise anteroposterior localization of the cysts, assessment of possible Descemet membrane penetration, and evaluation of endothelial invasion could not be performed.

At 1-year follow-up (50 weeks after referral), the globe was preserved, although dense corneal opacity and marked corneal thinning remained (Fig. 1F). Final visual acuity was hand motion, with intraocular pressure of 15 mmHg. Anterior segment optical coherence tomography (not shown) suggested marked thinning of the residual corneal layer after spontaneous sloughing; however, dense opacity and irregular residual stromal tissue precluded reliable quantitative pachymetry and detailed assessment of the anterior chamber, crystalline lens, and cataract status. The preserved globe contour, the absence of frank perforation or intraocular tissue prolapse after sloughing, and the absence of the Descemet membrane or corneal endothelium in the sloughed specimen suggested that posterior corneal structures were likely intact. However, this inference could not be confirmed directly histopathologically because the residual posterior cornea was not sampled. Immediate keratoplasty was deferred because of the high surgical risk, including marked corneal vascularization with a high risk of graft rejection, extremely thin residual corneal tissue after melting and sloughing, limited suture purchase, potential poor graft–host adhesion, and an uncertain anterior segment configuration. The overall chronology of the clinical and medication courses is summarized in Fig. 2A, B, and the sequential anterior segment photographs are shown in Fig. 3A–H.

## Discussion

AK can masquerade as other corneal or anterior segment disorders before typical clinical signs become evident [1, 2]. The established early clinical spectrum includes epithelial irregularity, superficial punctate keratitis, microcystic edema, radial keratoneuritis, multifocal stromal infiltrates, and ring infiltrates [1, 2]. Hsu et al. and Lakhani et al. reported AK with stromal edema, keratic precipitates, and anterior chamber inflammation mimicking corneal endotheliitis despite an intact corneal epithelium [3, 4]. The present case resembled these reports in its clinical presentation, but the initial endotheliitis-like diagnosis was based on clinical appearance rather than histopathologic confirmation of primary endothelial infection. The subsequent development of ring-like infiltration, stromal necrosis, and histopathologic confirmation of *Acanthamoeba* cysts in the sloughed stromal tissue supported the final diagnosis of AK with an atypical early presentation.

Although corneal endotheliitis-like presentations of AK have been reported [3, 4], keratic precipitates and anterior chamber inflammation should not be interpreted as definitive evidence of primary endothelial infection by *Acanthamoeba.* The examined specimen in the present case was not a keratoplasty specimen but a spontaneously sloughed necrotic stromal fragment. Because identifiable corneal epithelium, the Descemet membrane, and corneal endothelium were not observed in the specimen, precise anteroposterior localization of the cysts, assessment of possible Descemet membrane penetration, and evaluation of endothelial invasion were not possible. Nevertheless, several considerations argue against proven primary amoebic endothelial invasion. AK usually involves the corneal epithelium and stroma, and experimental and review data suggest that the Descemet membrane and corneal endothelium may limit intraocular extension of *Acanthamoeba* organisms [5, 6]. The generally recognized tissue involvement in AK, in which organisms are usually detected in the epithelium and stroma while the Descemet membrane often remains an important posterior barrier, also provides rationale for the use of deep anterior lamellar keratoplasty in selected medically nonresponsive AK cases with disease limited to tissues anterior to the Descemet membrane [7]. In our case, the subsequent course was dominated by progressive deep stromal necrosis, stromal melting, and sequestrum formation, and histopathology of the sloughed tissue demonstrated cystic structures consistent with *Acanthamoeba* within necrotic stromal tissue. Taken together, the initial keratic precipitates and anterior chamber inflammation are more plausibly interpreted as endotheliitis-like secondary inflammatory findings associated with occult or deep stromal AK, possibly with an immune-mediated component and modified by prior corticosteroid exposure rather than as direct amoebic invasion of the endothelium. However, because the Descemet membrane and corneal endothelium were not available for histopathologic evaluation, direct endothelial involvement cannot be completely excluded.

This distinction is clinically relevant because the absence of epithelial defects and radial keratoneuritis at referral contributed to the initial consideration of viral endotheliitis or anterior uveitis. As the clinical course evolved toward a more typical AK appearance, repeated diagnostic reassessment and antiamoebic therapy became necessary.

Corticosteroid exposure is an important clinical issue, but causal interpretation should be done with caution. Previous studies have associated corticosteroid use before AK diagnosis and longer symptom duration with poorer outcomes [8–10], and a recent systematic review and meta-analysis similarly suggested that prediagnosis corticosteroid use is associated with a lower likelihood of good visual outcome [11]. A Japanese clinical series has also described risk factors and clinical signs associated with severe AK [12]. In contrast, topical corticosteroids administered after antiamoebic therapy have not necessarily been associated with worse outcomes in selected cases [13]. In the present case, corticosteroid exposure may have modified the inflammatory phenotype and local host response. However, the mechanism underlying the destructive stromal course cannot be determined from a single case report. Diagnostic delay, deep stromal infection, organism-related factors, limited penetration of amoebicidal agents, and possible but unproven microbial interactions should all be regarded as plausible contributors rather than proven mechanisms.

Microbiological confirmation proved challenging in this case. Although corneal scraping was performed when AK was clinically suspected, repeated corneal scraping specimens showed no organisms on direct microscopy and were negative on bacterial and fungal cultures. PCR testing for *Acanthamoeba*, bacterial, and fungal DNA was performed once using a corneal scraping specimen, and all results were negative. Calcofluor white staining was not performed on the scraping specimens. Therefore, the diagnosis at that time was clinical, based on the evolving keratitis pattern and subsequent course. Histopathologic confirmation was eventually obtained from the spontaneously sloughed stromal tissue, which demonstrated multiple double-walled cystic structures consistent with *Acanthamoeba* cysts. Periodic acid-Schiff staining highlighted cyst wall-like structures, and no obvious fungal hyphae were identified in the examined hematoxylin and eosin or periodic acid-Schiff-stained sections. The negative scraping results may have reflected sampling limitations, the deep stromal location of organisms, or prior antimicrobial and anti-inflammatory treatment. Grocott methenamine silver, Gram, and calcofluor white staining were not performed on the histopathologic specimen; therefore, definitive histopathologic statements excluding fungal or bacterial coinfection were avoided.

The Japanese Clinical Practice Guidelines for Infectious Keratitis, 3rd edition, describes chlorhexidine or PHMB as first-line topical therapy for AK, while noting that Brolene (propamidine isethionate) and antifungal agents may be used adjunctively with biguanide disinfectants, although the evidence for their efficacy is limited; the efficacy of systemic antifungal therapy is also unclear [14]. In the present case, because effective therapeutic options for progressive AK were limited, the disease continued to worsen, and fungal or mixed microbial keratitis could not be excluded clinically, pimaricin, oral itraconazole, and later topical voriconazole were used for adjunctive empirical coverage rather than as standard antiamoebic therapy.

The antimicrobial regimen in this case should be interpreted in the context of diagnostic uncertainty during severe, progressive keratitis. Chlorhexidine 0.02% was used as the principal antiamoebic therapy. Pimaricin 1% ointment, oral itraconazole, and later topical voriconazole 1% were not intended to represent standard antiamoebic therapy. Rather, these agents were used as adjunctive empirical antifungal coverage because fungal keratitis or mixed microbial keratitis could not be completely managed clinically, particularly during periods of progressive dense whitish stromal infiltration and stromal melting. We therefore present the use of these antifungal agents for adjunctive empirical coverage based on clinical diagnostic uncertainty rather than as evidence of confirmed mixed infection or routine institutional practice.

Severe or advanced AK may be associated with serious inflammatory complications and the need for surgical intervention. *Acanthamoeba* sclerokeratitis has been described as a severe inflammatory complication requiring complex management [15, 16]. Keratoplasty series have also documented therapeutic or optical keratoplasty for medically uncontrolled disease, perforation, or severe thinning [17, 18], and a recent systematic assessment summarized severe complications in AK [19]. Recent reviews have also emphasized the diagnostic, pathogenetic, and therapeutic complexity of AK [20].

Mixed microbial keratitis is another consideration in severe AK. In the present case, bacterial and fungal cultures from corneal scraping were negative, and no microbiological evidence of bacterial or fungal coinfection was identified. Recent studies have suggested that *Acanthamoeba* may interact with other ocular pathogens, including intracellular bacteria, and that such interactions may influence disease severity [21, 22]. These mechanisms were not assessed in our patient because additional molecular or microbiome analyses were not performed. Therefore, potential microbial interactions are discussed only as a broader consideration in severe or atypical AK rather than as an established mechanism in this case.

The severe stromal melting and spontaneous necrotic sloughing observed in this case should be interpreted in the context of previously reported destructive complications of AK (Table 2). Advanced AK has been associated with medically nonresponsive ulceration, marked corneal thinning, descemetocele formation, perforation, and keratoplasty-level destruction. Prior case reports and clinical series have described surgical intervention for uncontrolled infection, perforation, or severe thinning, including cases complicated by fungal coinfection, trauma, post-procedural epithelial compromise, or refractory intraocular extension [23–37]. However, spontaneous sloughing of necrotic corneal tissue as a sequestrum, with histopathologic confirmation of multiple *Acanthamoeba* cysts within the sloughed deep stroma, is rarely documented. Therefore, the present case adds a clinicopathologic correlate to the spectrum of severe AK by showing that destructive stromal disease may manifest not only as perforation or need for keratoplasty but also as spontaneous necrotic stromal sloughing.

**Table 2:** Severe or destructive *Acanthamoeba* keratitis reports compared with the present case

Study	Study type/cases	Severe/destructive features	Corticosteroid exposure	Necrosis/stromal destruction and outcome/surgery	Relevance to present case
Mohammadpour et al. [23]	Case report	Soft contact lens wearer with recalcitrant AK; ring infiltration and deep stromal invasion progressed to spontaneous total necrosis/dislodgment of the cornea, iris, and crystalline lens with exposure of the vitreous hyaloid face.	Topical corticosteroid use before proper diagnosis was described.	Urgent debridement, anterior vitrectomy, tectonic PK, AMT, and temporary tarsorrhaphy were performed. The graft remained clear for 4 years, but the eye became phthisic and final VA remained light perception.	Closest comparator for extreme tissue necrosis/melting and prediagnosis corticosteroid exposure; however, tissue loss involved total anterior-segment dislodgment rather than a sloughed stromal sequestrum containing cysts.
Vemuganti et al. [24]	Histopathologic study of corneal tissues from AK	Histopathologic analysis of keratocyte loss and stromal necrosis in AK.	Not the primary focus.	Inflammation and necrosis in the anterior two-thirds of the stroma; cysts were more common in deeper stroma with minimal or no inflammatory response. Corneal tissues from keratoplasty/evisceration cases were analyzed.	Supports the pathologic plausibility of stromal necrosis and deeper stromal cyst distribution in AK, but does not describe spontaneous sloughed sequestrum.
Kitzmann et al. [25]	Retrospective keratoplasty series; 31 eyes (22 therapeutic, 9 optical keratoplasties)	Medically unresponsive AK requiring keratoplasty; 82% of therapeutic keratoplasty eyes had ring infiltrate in the comparative summary.	Case-level corticosteroid details were not the main focus.	Severe active keratitis requiring corneal replacement; necrotic sloughing was not specifically described. Therapeutic keratoplasty was associated with high morbidity, recurrent infection, and regrafting in the keratoplasty literature.	Establishes keratoplasty-level severe AK, but differs from the present case because spontaneous stromal sloughing provided diagnostic tissue without immediate keratoplasty.
Robaei et al. [17]	Retrospective keratoplasty series; 50/196 AK eyes (26 therapeutic, 24 optical keratoplasties)	Therapeutic keratoplasty was performed predominantly for corneal perforation or uncontrolled disease; the keratoplasty group had longer diagnostic delay and more scleritis.	Topical corticosteroid use before diagnosis was documented in 16/22 therapeutic keratoplasty eyes (72.7%) and 21/23 optical keratoplasty eyes (91.3%) with available data.	Perforation/keratoplasty-level destruction; spontaneous necrotic stromal sequestrum was not described. Ten patients (20%) required repeat keratoplasty; recurrent infection occurred in 1 therapeutic keratoplasty eye; final VA < 20/200 occurred in 14/26 therapeutic versus 2/24 optical keratoplasty eyes.	Useful comparator for advanced AK requiring corneal transplantation; the present case differs because the sloughed necrotic stroma itself provided histopathologic confirmation and keratoplasty was deferred.
Roozbahani et al. [26]	Retrospective TPK case series; 12/63 AK eyes required therapeutic penetrating keratoplasty	Indications for TPK were medically nonresponsive ulcer in 7 eyes (58%), perforated ulcer in 3 eyes (25%), and significant thinning/descemetocele in 2 eyes (17%).	Topical corticosteroid use before AK diagnosis was present in 8/12 TPK eyes (67%).	Nonresponsive ulceration with deepening infiltration and significant thinning; spontaneous necrotic sequestrum was not described. Post-TPK complications included graft failure (75%), cataract (50%), glaucoma surgery (17%), and AK reactivation in 1 eye (8%). Delayed antiamoebic treatment (>25 days) and poorer presenting vision independently predicted TPK.	Supports marked thinning, descemetocele/perforation, and TPK as recognized severe AK outcomes; the present case differs by spontaneous sloughing of infected necrotic stroma rather than planned emergency TPK.
Zhang et al. [27]	Retrospective severe AK therapeutic keratoplasty series; 59 eyes (36 PK, 23 LK), all stage 3	Severe AK deteriorating after at least 1 week of antiamoebic therapy; PK was selected for full-thickness infection and LK for partial-depth infection.	Topical corticosteroid use before diagnosis was a recurrence risk factor after LK (*p* = 0.040) and after PK (*p* = 0.045); hypopyon was also a recurrence risk factor after LK (*p* = 0.009).	Severe stromal disease requiring PK/LK; postoperative recurrence manifested as greyish-white recipient-bed infiltration, anterior chamber inflammation, graft edema, and keratic precipitates. Necrotic sequestrum was not described. Globe salvage was 91.7% after PK and 91.3% after LK; final VA was ≥ 20/60 in 14 PK eyes (38.9%) and 15 LK eyes (65.2%); recurrence occurred in 10 eyes.	Provides a modern severe AK surgical comparator. The present case was managed without immediate keratoplasty because of high surgical risk, despite severe melting and thinning.
Robaei et al. [9]	Retrospective cohort on prediagnosis corticosteroids; 196 AK eyes, 174 analyzable for steroid/outcome data	Suboptimal outcome was defined as final VA ≤ 20/80, corneal perforation, or need for keratoplasty.	Topical corticosteroid use before diagnosis was present in 87/174 eyes (50%) and was independently associated with suboptimal outcome (OR, 3.90; 95% CI, 1.78–8.55).	Perforation/keratoplasty were included as outcome categories; necrotic sloughing was not described. Patients exposed to corticosteroids before diagnosis more often required keratoplasty than unexposed patients (37/87 [42.4%] vs 8/87 [9.2%]).	Supports discussing corticosteroid exposure as a risk factor for poor outcome, while not proving direct causality for stromal necrosis in the present single case.
Carnt et al. [10]	Retrospective cohort; 194 AK eyes	Bad outcome was defined as corneal perforation, keratoplasty, other ocular surgery, AAT ≥ 10.5 months, or final VA ≤ 20/80; bad outcomes occurred in 93 eyes (48%).	Topical corticosteroid use before AAT was more common in bad outcomes than better outcomes (56/83 [67.5%] vs 35/98 [35.7%]) and was an independent risk factor in multivariable analysis.	Severe inflammatory complications were defined as scleritis and/or stromal ring infiltrate; hypopyon was also more frequent in bad outcomes (19/83 [22.9%] vs 8/100 [8.0%]). Necrotic sequestrum was not described.	Supports a multifactorial interpretation of destructive AK, involving diagnostic delay, pre-AAT corticosteroid exposure, and severe inflammation rather than a single proven mechanism.
Carnt et al. [13]	Retrospective cohort on topical corticosteroids after antiamoebic therapy	Assessed outcomes after topical corticosteroids were used with established antiamoebic therapy.	Post-AAT corticosteroid use was evaluated separately from prediagnosis/pre-AAT use.	Necrotic sloughing was not described. Topical corticosteroids after antiamoebic therapy were not necessarily associated with worse outcomes in selected cases.	Helps balance interpretation of corticosteroid effects; timing of corticosteroid exposure is clinically important.
Wouters et al. [28]	Retrospective study of topical corticosteroids in AK	Evaluated outcomes associated with corticosteroid timing.	Corticosteroid use before antiamoebic therapy was associated with suboptimal visual outcome and a higher risk for PK.	Necrotic sloughing was not described. The report highlights the timing-dependent effect of corticosteroid exposure.	Supports cautious interpretation of corticosteroid exposure as a risk factor, not a proven direct cause of stromal destruction.
Rama et al. [29]	Case report after corneal crosslinking and bandage contact lens use	Rapid post-CXL AK with initial descemetocele, corneal melting by day 6, and perforation by day 11.	Chloramphenicol/betamethasone ointment and oral prednisone 25 mg/day were prescribed before referral; topical NSAID exposure was also discussed.	Corneal smear was positive for *Acanthamoeba*. A 10.0-mm therapeutic PKP was performed; the corneal button showed stromal necrosis and severe granulocytic infiltrate but no organisms. At 2 months, BCVA was 20/200, improving to 20/40 with pinhole, and the graft was clear without recurrence.	Demonstrates rapid melting/perforation in AK with epithelial compromise and corticosteroid/NSAID exposure; differs from the present case because the clinical setting was post-CXL and the removed button did not show organisms.
Froumis et al. [30]	Case report; bilateral contact lens-related AK complicated by fungal keratitis	Protracted bilateral AK; the left eye developed corneal ulceration, perforation, and mature cataract.	Initial cyclosporin A and prednisolone acetate were used for presumed herpes simplex virus keratitis.	PK, lensectomy, and vitrectomy were performed. Histopathology showed extensive lamellar stromal necrosis with double-walled empty cystic structures and branching hyphae; culture grew Scedosporium apiospermum. The course required repeat PK and was complicated by retinal detachment; final VA was counting fingers in both eyes.	Documents stromal necrosis with cystic structures in a keratoplasty specimen and fungal coinfection, but does not describe spontaneous sloughing of a cyst-containing stromal sequestrum.
Rumelt et al. [31]	Case report; sewage-contaminated traumatic corneal coinfection	*Acanthamoeba* and Scedosporium apiospermum coinfection after contaminated injury; ulcer progressed to entire corneal involvement, severe thinning, and complete perforation with partial crystalline lens expulsion.	Initial broad-spectrum therapy included topical corticosteroids for presumed bacterial infection; the authors advised withholding corticosteroids until causative organisms are identified.	Scraping/biopsy showed fungal elements and *Acanthamoeba* cysts. After perforation, urgent PK with intracapsular extraction of the partially expelled lens and vitrectomy was performed. The corneal button was necrotic but devoid of organisms; the graft remained clear and corrected VA improved to 20/70.	Supports the role of mixed infection, trauma, diagnostic delay, and corticosteroid exposure in severe thinning/perforation; tissue loss was perforative rather than a spontaneously sloughed stromal sequestrum containing cysts.
Lee et al. [32]	Case report; contact lens-related *Acanthamoeba*-Fusarium coinfection	Early stromal keratolysis progressed to severe stromal keratolysis, layered hypopyon, impending corneal melt, and total corneal opacification.	Tobramycin/dexamethasone was used before referral; a dispensing error caused additional neomycin/polymyxin B/dexamethasone exposure, for a total of 8 days of topical corticosteroid use.	Therapeutic limbus-to-limbus PK was performed for impending melt. Histopathology confirmed *Acanthamoeba* cysts and fungal elements. Vision declined to light perception, and two repeat PKs were needed; final BCVA was 20/400 due to retinal and optic nerve damage from secondary glaucoma.	Shows severe keratolysis with documented cysts and fungal elements after corticosteroid exposure; differs from the present case because destruction required therapeutic keratoplasty rather than spontaneous diagnostic sloughing.
Tavassoli et al. [33]	Case report; refractory AK with intraocular spread	Persistent/recurrent AK with hypopyon and scleritis progressed to severe necrotic keratitis with almost complete corneal melt.	Topical dexamethasone was started after initial improvement; after PKP, further immunosuppression including prednisolone/tacrolimus and intravenous methylprednisolone was used before persistent infection was recognized.	Therapeutic PKP showed *Acanthamoeba* throughout the host stroma. Persistent infection was confirmed by AC tap, corneal scrape, and biopsy; enucleation was ultimately required. Histopathology detected A. polyphaga and A. castellanii in the vitreous but not retina, choroid, or optic nerve.	Represents an extreme refractory AK comparator with necrotic melt and intraocular spread; more destructive than the present case and ultimately required enucleation.
Domínguez-Serrano et al. [34]	Case report; first-trimester pregnant contact lens wearer	A melting ulcer appeared 3 months after initiation of antiamoebic therapy; confocal microscopy ruled out trophozoites, suggesting an immunologic rather than active infectious etiology.	Gentamicin/dexamethasone was used before diagnosis; later preservative-free dexamethasone was used after the melting ulcer was considered immunologic.	Amebicidal therapy was discontinued because of corneal toxicity; treatment with a regenerating agent (Cacicol) closed the persistent epithelial defect within 1 week. BCVA was 20/20 with a paracentral stromal scar.	Useful counterpoint showing that melting may also occur after apparent microbiologic control and may be immunologic/toxic rather than direct amoebic tissue invasion; less destructive than the present case.
Nasef et al. [35]	Retrospective series; 44 eyes from 42 AK patients	Late-presenting AK included corneal melting; surgery was required in 5 eyes (11.3%) because of progressive corneal thinning and perforation.	Prior topical corticosteroids were reported in 11/44 eyes (25%).	Four eyes underwent AMT and one eye underwent tectonic keratoplasty. Recurrent lesions were not recorded; 17 patients (38.6%) had final BCVA of 0.2 or better. Culture was reported to be more sensitive in late presentation with corneal melting.	Provides a clinical-series comparator showing that progressive thinning/perforation and tectonic surgery are recognized severe AK outcomes; spontaneous necrotic sequestrum was not described.
Megha et al. [36]	Prospective/4-year tertiary-care review; 11 AK patients	Severe ulcers (>5 mm and/or >2/3 stromal involvement) were seen in 5/11 patients; perforation/corneal melt occurred in 3/11 (27.3%).	None of the patients received corticosteroids before presentation.	The 3 perforation/melt cases underwent therapeutic keratoplasty; one had persistent infection after keratoplasty and developed phthisis bulbi. Seven patients healed with vascularized corneal scarring, but visual outcomes were often limited.	Shows that trauma-associated and delayed AK can progress to perforation/melt even without pre-presentation corticosteroids; no spontaneous cyst-containing sequestrum was described.
Atighehchian et al. [37]	Case report; fungal keratitis complicating AK diagnosis	Unresponsive ulcer initially treated as HSV/fungal keratitis progressed with stromal edema, necrosis, hypopyon, significant stromal keratolysis, and limbal involvement.	Prediagnosis corticosteroid exposure was not described in the case narrative.	Early T-PKP with cryotherapy and intracameral voriconazole was performed. Histopathology and PCR confirmed *Acanthamoeba* cysts and Fusarium; topical/systemic voriconazole and PHMB were continued. At 6 months, BCVA was 1.0 logMAR with no recurrent graft infiltration.	Supports the need to consider AK in progressive fungal-appearing ulcers and demonstrates keratolysis requiring T-PKP with histopathologic cyst confirmation; spontaneous stromal sloughing was not reported.
Present case	Case report	Severe corneal melting with spontaneous sloughing of necrotic corneal tissue as a sequestrum.	Topical and systemic corticosteroids were used before clinical AK diagnosis; a prolonged taper is documented in Table 1.	H&E and PAS staining showed necrotic corneal stroma with inflammatory cell infiltration and multiple double-walled or cyst wall-like structures within the sloughed necrotic stromal matrix. The globe was preserved, final VA was hand motion, and immediate keratoplasty was not planned because of high surgical risk.	Adds clinicopathologic documentation of spontaneous necrotic stromal sloughing containing *Acanthamoeba* cysts, distinct from typical perforation/keratoplasty endpoints.

This case highlights several practical considerations in the diagnostic evaluation of AK. In contact lens wearers with stromal edema, keratic precipitates, and anterior chamber inflammation, AK should remain in the differential diagnosis even when the corneal epithelium is initially intact. When presumed viral endotheliitis or anterior uveitis follows an atypical or progressive course, repeated diagnostic reassessment is important before continuing or escalating corticosteroid therapy. Depending on local availability, repeat corneal scraping, calcofluor white staining of scraping specimens, repeat PCR testing, and in vivo confocal microscopy may be considered as adjunctive diagnostic approaches. Early anterior segment imaging or serial slit-lamp photographic documentation may facilitate detection of progressive stromal infiltration, stromal thickening, or melting, though these approaches are adjunctive and do not replace microbiological or histopathologic confirmation.

## Conclusions

AK may mimic corneal endotheliitis with stromal edema, keratic precipitates, Descemet membrane folds, and anterior chamber inflammation before typical epithelial or perineural findings become clinically evident. A notable feature of this case was the documented clinicopathologic sequence: negative scraping-based microbiological tests during the active course, progression to severe corneal melting with spontaneous sloughing of necrotic corneal tissue as a sequestrum, and subsequent hematoxylin and eosin and periodic acid–Schiff confirmation of multiple double-walled cysts within the sloughed necrotic stroma. This case highlights the importance of repeated diagnostic reassessment in contact lens wearers with atypical endotheliitis-like keratitis and provides a clinicopathologic example of severe AK manifesting as necrotic stromal sequestration and spontaneous sloughing.

## Data Availability

No datasets were generated or analysed during the current study.

## Abbreviations

AK: *Acanthamoeba* keratitis
H&E: Hematoxylin and eosin
PAS: Periodic acid–Schiff
PCR: Polymerase chain reaction
